# MASLD and Pancreatic Diseases: From Shared Mechanisms to Clinical Implications and Future Directions

**DOI:** 10.3390/nu18152480

**Published:** 2026-07-31

**Authors:** Jesús Rivera-Esteban, Belén Agudo, Beatrice Patrizi, Alejandra Manzur, Daniel de la Iglesia, Víctor Valverde, Neus García Ferris, Carlos Esteban, Elena Santos, Fernando Pons, Marta Hernández-Conde, Javier Abad, Mariano González-Haba, José Luis Calleja

**Affiliations:** 1 Department of Gastroenterology and Hepatology, Hospital Universitario Puerta de Hierro Majadahonda, Instituto de Investigación Sanitaria Puerta Hierro-Segovia Arana (IDIPHISA), 28222 Madrid, Spain; bagucast@gmail.com (B.A.); alejandramanzur95@gmail.com (A.M.); danieldelaiglesiagarcia@gmail.com (D.d.l.I.); v.valverde@idiphim.org (V.V.); neusgferris@gmail.com (N.G.F.); carlosestebanfz@yahoo.es (C.E.); elenasantosperez8@gmail.com (E.S.); fponsr@hotmail.com (F.P.); marta.hernandez.conde@gmail.com (M.H.-C.); javiabad83@gmail.com (J.A.); marianoghr@gmail.com (M.G.-H.); joseluis.calleja@uam.es (J.L.C.); 2 School of Medicine, Universidad Autónoma de Madrid, 28049 Madrid, Spain; 3 Internal Medicine and Centre for Hemochromatosis and Hereditary Liver Diseases, AOU Policlinico, University of Modena and Reggio Emilia, 41126 Modena, Italy; beatricep17.bp@gmail.com

**Keywords:** metabolic dysfunction-associated steatotic liver disease, insulin resistance, pancreatic diseases, cardiometabolic risk factors

## Abstract

Metabolic dysfunction-associated steatotic liver disease (MASLD) is increasingly recognised as a systemic disorder with clinically relevant extrahepatic manifestations. Pancreatic diseases have gained particular attention in this context, as they share key metabolic and inflammatory mechanisms with MASLD and may influence each other’s natural history and clinical outcomes. Beyond shared cardiometabolic risk factors, emerging evidence suggests that MASLD may actively contribute to pancreatic dysfunction and disease progression, supporting a bidirectional liver–pancreas relationship. Recognition of this association is essential to improve risk stratification, diagnostic assessment, and clinical management. This review summarises the current evidence linking MASLD with pancreatic disorders, focusing on acute and chronic pancreatitis, pancreatic cancer, and fatty pancreas. We examine shared pathophysiological pathways and discuss how these interactions may affect disease progression, nutritional status, diagnostic strategies, and therapeutic decision-making. We also address key clinical challenges, including alcohol assessment and MASLD–MetALD misclassification, highlight current knowledge gaps, and provide scenario-specific recommendations.

## 1. Introduction

Metabolic dysfunction-associated steatotic liver disease (MASLD) has become the leading cause of chronic liver disease and represents a major global healthcare challenge. It affects approximately one-third of adults in Western countries, and its prevalence is projected to increase markedly, potentially reaching 50% worldwide by 2040 [1].

Rather than representing an isolated hepatic condition, MASLD is recognised as the hepatic manifestation of the metabolic syndrome (MetS). It is closely associated with major cardiometabolic risk factors (CMRF), including obesity and type 2 diabetes (T2D), as well as systemic inflammation and endocrine dysfunction. These factors contribute to disease progression, the development of liver-related outcomes and extrahepatic complications [2,3].

Within this context, pancreatic disorders have gained increasing attention, as they share several fundamental pathophysiological mechanisms with MASLD, including chronic inflammation, oxidative stress, and metabolic dysregulation [4]. This common ground supports a bidirectional relationship, with accumulating evidence indicating that pancreatic dysfunction may play an active role in modulating MASLD progression and shaping clinical outcomes, although the underlying mechanisms remain incompletely understood.

Here, we provide a comprehensive overview of the current evidence linking MASLD with pancreatic disorders. We focus on major and emerging conditions, such as acute and chronic pancreatitis, pancreatic cancer, and fatty pancreas disease, and provide insights about less frequent, albeit relevant disorders. In addition, we examine the key shared pathophysiological pathways and consider how these interactions may influence disease progression, diagnostic strategies, and therapeutic decision-making in both hepatic and pancreatic disease. Finally, we discuss the related nutritional consequences and management, and the current main knowledge gaps and our vision about further research in the field.

## 2. Material and Methods

We conducted a systematic literature search addressing the primary focus of this review: the association between MASLD and pancreatic disorders. SCOPUS, MEDLINE (through Ovid), and Scielo databases were electronically searched. The search combined free-text terms and controlled vocabulary related to MASLD, liver cirrhosis and pancreatic disorders, including MASLD OR liver disease AND pancreatic cancer OR acute pancreatitis OR chronic pancreatitis. Titles and abstracts were independently screened by JRE and BA, and full-text articles published in English or Spanish were assessed for eligibility if they specifically examined the relationship between MASLD and the corresponding pancreatic disorder. The remaining sections of the review were informed by a non-systematic narrative search.

## 3. MASLD as a Systemic Disease

The systemic nature of MASLD is reflected in its conceptual framework and nomenclature. Under the current umbrella of steatotic liver disease (SLD), which encompasses a range of conditions characterised by hepatic fat accumulation, the terminology has evolved from the hepatic-centered, exclusionary and potentially stigmatising term non-alcoholic fatty liver disease to a more inclusive and metabolically driven concept-metabolic dysfunction-associated steatotic liver disease [5]. This shift reflects both the central role of steatosis and the pivotal contribution of metabolic dysfunction to its pathogenesis and progression.

Cardiometabolic risk factors (obesity, T2D, dyslipidemia, and hypertension) are the main drivers for MASLD onset and progression, promoting the development of both liver and non-liver-related events [6]. In fact, cardiovascular disease and malignancies are the leading causes of mortality in the entire MASLD population [7].

Importantly, compared with other liver disease etiologies, MASLD has been associated with a higher incidence of hepatocellular carcinoma (HCC) in pre-cirrhotic stages [8,9] and extrahepatic malignancies, including colorectal, pancreatic, breast, and renal cancers [10]. These findings point to a broader systemic oncogenic potential that extends beyond the stage of liver disease.

The pathophysiology of MASLD is highly complex and involves interconnected processes such as chronic inflammation, insulin resistance, endothelial dysfunction, and a range of genetic and epigenetic factors, including gut dysbiosis [11,12]. These mechanisms contribute not only to hepatic injury but also to the development of a broad spectrum of extrahepatic manifestations, underscoring the systemic nature of the disease (Table 1).

Notably, endocrine dysfunction is well documented in patients with MASLD, affecting multiple organs, including the thyroid (e.g., hypothyroidism) [13] and the gonads (e.g., male hypogonadism and polycystic ovary syndrome) [14]. Pancreatic involvement is of particular importance, as MASLD appears to contribute to both acute and chronic pancreatic disorders, which carry significant morbidity and mortality, while also impairing endocrine and exocrine function. Conversely, pancreatic dysfunction may influence MASLD severity and its natural history [15].

## 4. Pancreatic Diseases and MASLD

Pancreatic diseases constitute a major cause of hospitalisation and reduced quality of life among gastrointestinal disorders. Recent global estimates indicate that acute and chronic pancreatitis affected an estimated 2.75 million individuals worldwide and were responsible for approximately 122,000 deaths [16], whilst pancreatic cancer alone accounted for over 500,000 deaths worldwide in 2021–2022, ranking as the sixth leading cause of cancer-related mortality [17].

The liver and pancreas are closely interconnected through their central roles in metabolic homeostasis, a relationship that becomes particularly relevant in the context of metabolic disorders such as MASLD. In addition, MASLD, together with CMRF, genetic and epigenetic modifiers, may promote pancreatic disorders and contribute to pancreatic dysfunction [12].

In the following sections, we summarise the current understanding of the pathophysiological mechanisms linking MASLD and pancreatic disorders, along with the available evidence on pancreatic events in this population. Importantly, interpretation of these associations requires careful adjustment for shared confounders, causality, and surveillance/survival bias.

## 5. Pathophysiological Relationship Between MASLD and Pancreatic Disorders

The liver and pancreas are metabolically active organs with key endocrine and exocrine functions. Insulin resistance, metabolic stress and gut dysbiosis promote systemic inflammation and organ crosstalk through the gut–liver–pancreas axis, influencing liver and pancreatic disease development [12,18].

Insulin resistance (IR) is the main driver of MetS and related disorders, and its pathophysiology involves different organs. Reduced glucose uptake in peripheral tissues increases circulating glucose and free fatty acids (FFAs). In the liver, IR impairs gluconeogenesis, promotes lipogenesis and enhances hepatic steatosis. Finally, compensatory hyperinsulinemia places stress on pancreatic β-cells, leading to dysfunction and progressive failure, which further exacerbates IR and metabolic imbalance [19].

MASLD and advanced chronic liver disease typically present chronic low-grade inflammation, characterised by increased pro-inflammatory cytokines (i.e., tumour necrosis factor-α or interleukin-6), macrophage and inflammasome activation. In both the liver and the pancreas, these processes promote stellate cell activation, fibrosis, and structural remodelling. Consequently, disruption of acinar, ductal, and islet architecture may lead to progressive impairment of endocrine and exocrine pancreatic function. Liver-derived mediators may further modulate pancreatic function: fetuin-A, fetuin-B, and selenoprotein P have been associated with IR and cellular stress, whereas FGF21 may enhance insulin sensitivity and exert protective effects on pancreatic β-cells [20,21,22,23].

In addition, gut dysbiosis increases intestinal permeability, facilitating endotoxins and microbe-derived metabolites to reach splanchnic organs, such as the liver and the pancreas, through portal circulation. The pathogenic role of gut dysbiosis in MASLD is well established, contributing to disease onset and progression, and has prompted the evaluation of gut-targeted interventions such as dietary modification and faecal microbiota transplantation [24]. By contrast, evidence linking dysbiosis to pancreatic disorder, albeit biologically plausible, is preliminary and warrants further investigation [25].

The main mechanisms of acute pancreatic injury involve calcium and organelle homeostasis, along with trypsin activation. Bile acids, alcohol metabolites, secretagogue hyperstimulation, and ductal or mechanical stress induce a sustained cytosolic calcium overload by enhancing ER calcium release, overwhelming ATP-dependent extrusion mechanisms. The resulting activation of calcineurin and loss of cellular energy homeostasis trigger mitochondrial permeability, ATP depletion, ER stress and epithelial junction disruption. At the same time, impaired autophagic–lysosomal processing causes vacuole accumulation and favours digestive zymogens interaction with lysosomal hydrolases, promoting intracellular trypsinogen activation. These events reinforce one another, ultimately driving acinar-cell necrosis [26,27].

In this scenario, the metabolic milieu frequently accompanying MASLD may increase susceptibility and severity of pancreatic injury by increasing triglyceride-rich lipoproteins and intrapancreatic fat availability that can be hydrolysed by pancreatic lipases into toxic free fatty acids. These lipids directly disturb cellular and mitochondrial membranes, aggravate calcium overload, impair immune-cell phagocytosis, and promote pancreatic and distant-organ injury. Acinar necrosis subsequently releases activated proteases, ROS, mitochondrial DNA, and other damage-associated molecular patterns, activating NF-κB, inflammasome signalling and pro-inflammatory macrophages [28]. Endothelial injury, impaired microcirculation, hypoxia, and increased gut permeability further amplify inflammation and favour bacterial translocation.

Ultimately, disease evolution depends on the balance between injury and repair. Pancreatic injury resolution requires clearance of necrotic tissue, revascularisation, and a shift from inflammatory to reparative macrophage phenotypes. When these mechanisms fail, or injury recurs, persistent cytokine signalling activates pancreatic stellate cells and promotes fibrosis, chronic pancreatitis, and organ dysfunction [29]. This provides a mechanistic basis for a bidirectional liver–pancreas axis, whereby MASLD aggravates pancreatic injury, while pancreatic dysfunction further worsens IR, nutrient handling, and hepatic metabolic stress.

## 6. Acute Pancreatitis

Acute pancreatitis (AP) is the leading gastrointestinal cause of acute hospital admission, with an annual incidence of 29–38 per 100,000 individuals [30]. Although the majority of patients experience a mild, self-limiting disease with a <1% mortality rate, approximately 20–30% develop moderately severe or severe AP, characterised by local complications (i.e., peripancreatic fluid collections or necrosis) and/or persistent organ failure, with mortality rates up to 10–30% [31,32]. Regarding AP etiology, biliary gallstones remain the most common cause in Western countries, accounting for approximately 45% of cases, followed by alcohol abuse and hypertriglyceridemia [33].

The specific prevalence of MASLD in AP patients is not fully established. However, Váncsa et al. reported its presence in an estimated 39% of patients [34].

Although MASLD has been independently associated with biliary gallstones [35,36], the excess risk of acute biliary pancreatitis appears modest [15] in the MASLD population. In contrast, observational studies suggest that non-biliary aetiologies of AP, including alcohol and hypertriglyceridemia-related disease, are also overrepresented amongst MASLD patients [34,37]. Supporting this, non-biliary was the most common cause of AP in a biopsy-proven MASLD cohort.

Hypertriglyceridaemia, a key metabolic feature in this population, arises from impaired clearance of triglyceride-rich lipoproteins and increased hepatic VLDL production. The resulting elevation in circulating triglycerides and FFAs promotes pancreatic lipotoxicity and inflammation, potentially triggering AP, while also contributing to hepatic steatosis and steatohepatitis.

Moreover, an endoscopic ultrasound-based study comprising more than 300 patients reported a higher prevalence of fatty pancreas (FP) among individuals with a history of AP compared with those without [38], with pancreatic steatosis independently associated with a history of AP and hyperlipidemia. Although these findings do not establish causality, they support the hypothesis that FP may represent an intermediate metabolic phenotype linking MASLD, dyslipidemia and non-biliary AP.

Regarding severity, MASLD has been associated with higher odds of systemic inflammatory response syndrome, organ failure and mortality in the context of AP [39]. A meta-analysis including ten studies and 6436 individuals showed that MASLD patients present higher rates of moderate-to-severe and necrotising pancreatitis and mortality [40]. Importantly, these adverse outcomes appear to be driven by the underlying metabolic burden, as MASLD severity and the accumulation of CMRF demonstrate a dose-dependent relationship with AP severity compared to those patients metabolically healthy [37].

Overall, AP-related etiology and severity in MASLD patients are shaped primarily by the underlying metabolic context. The higher prevalence of non-biliary causes and adverse outcomes may reflect the greater burden of obesity, visceral adiposity, T2D, hypertriglyceridaemia, alcohol and tobacco exposure rather than a direct effect of MASLD itself. However, important potential confounders (i.e., medication use, glycaemic control, AP aetiology, centre expertise and disease management) are incompletely captured in most studies. Although current evidence supports a link between MASLD and a more severe AP phenotype, prospective studies with standardised hepatic and metabolic phenotyping are needed to clarify causality and determine whether MASLD provides prognostic information beyond its accompanying cardiometabolic burden.

## 7. Chronic Pancreatitis

Chronic pancreatitis (CP) is a progressive inflammatory disorder characterised by parenchymal and ductal damage, leading to fibrosis and exocrine and endocrine insufficiency when >90% of pancreatic tissue is lost. Annual incidence ranges from 5 to 14.4 cases per 100,000 population per year [41], and its etiology is multifactorial, reflecting the interplay between several pathogenic factors. Chronic alcohol and tobacco use are the main environmental drivers, acting synergistically to promote persistent injury and fibrogenesis. However, CP may also arise from ductal obstruction, genetic variants affecting pancreatic function, autoimmune mechanisms, or exposure to toxics [42]. In addition, CP represents a major risk factor for pancreatic ductal adenocarcinoma, justifying risk stratification and surveillance strategies in this population [43].

CP commonly coexists with CMRF. T2DM is the most frequent comorbidity, affecting approximately 26.3% of patients with CP, whilst 21.5% and 16.7% will suffer hypertension and dyslipidemia, respectively [44].

Several studies have examined the association of CP and liver diseases. Although the coexistence of CP and cirrhosis appears to be uncommon [45], the overall prevalence of SLD remains unclear. A population-based study reported a MASLD prevalence of 12.4% amongst CP patients [44], albeit surgical series with histological data suggest a higher burden of subclinical liver involvement, with hepatic abnormalities observed in up to 45% of cases, including macrovesicular steatosis in approximately 30% and fibrosis in around 15% [46].

Supporting a bidirectional association, a population-based cohort study of 8563 Swedish adults with biopsy-confirmed MASLD demonstrated a significantly higher incidence of CP compared with individuals without MASLD (0.54 per 1000 vs. 0.12 per 1000 person-years), with risk further increasing in the presence of MASLD-related advanced fibrosis or cirrhosis [15]. Although the biopsy-based and sibling-controlled design reduces exposure misclassification and some shared familial confounding, residual non-shared environmental and time-varying confounding cannot be excluded.

Overall, these findings support an association between MASLD and chronic pancreatitis, while also suggesting that hepatic involvement in patients with CP is more commonly characterised by steatosis than by advanced liver disease. MASLD may therefore represent a frequent but under-recognised entity in this setting, reflecting shared metabolic risk factors and the potential systemic consequences of pancreatic exocrine and endocrine insufficiency. Prospective longitudinal studies with serial hepatic and pancreatic phenotyping are needed to establish temporality and clarify the direction of this relationship.

## 8. Pancreatic Insufficiency

Pancreatic exocrine and endocrine insufficiency result from a massive loss of functional pancreatic tissue secondary to pancreatic disease, cancer, or pancreatic surgery [47,48,49].

Evidence regarding pancreatic exocrine insufficiency (PEI) and type 3c diabetes mellitus (T3cDM) in MASLD remains scarce, and the nature of this association has not yet been established. Preliminary data from Boga et al., in a cohort of biopsy-proven MASLD, showed significantly reduced faecal elastase-1 levels compared with controls, with MASLD independently associated with PEI after adjustment for age, sex, and diabetes mellitus [50]. However, these findings should be interpreted cautiously, as PEI was defined solely by low faecal elastase-1, without confirmation of maldigestion or additional functional testing [51].

## 9. Pancreatic Cancer

Pancreatic cancer represents the 12th most common cancer worldwide and the 6th leading cause of cancer-related death [52]. Pancreatic ductal adenocarcinoma (PDAC), which accounts for 90% of all pancreatic malignancies, is strongly linked with alcohol, tobacco, T2D, obesity, CP and genetic mutations [53].

MASLD is associated with an increased risk of extrahepatic malignancies (Table 1). In PDAC, large epidemiological studies suggest a higher incidence in MASLD patients, an association persistent after adjustment for metabolic comorbidities, smoking, alcohol use, and pre-existing pancreatic disease [54,55]. A large nationwide Korean study with over 8 million adults reported an obesity-independent increased risk of pancreatic cancer in individuals with MASLD, with a linear association according to steatosis severity [56]. Similarly, a Swedish biopsy-based cohort demonstrated a higher incidence of pancreatic cancer among MASLD subjects, with a twofold increased risk, already evident in simple steatosis and most pronounced in non-fibrotic MASH [15].

Moreover, it has been suggested that non-obese (lean) MASLD patients have a higher risk of hepatocellular, colorectal, and pancreatic cancers compared with overweight MASLD, underscoring the oncogenic relevance of the liver disease, especially at advanced stages, beyond adiposity alone [57]. In this line, experimental models suggest that MASLD may facilitate PC progression and metastasis through macrophage-mediated mechanisms; however, the relevance and magnitude of this effect in humans remain uncertain [58].

Tobacco represents a key risk factor for liver disease progression, malignancy and pancreatic disorders [59]. Smoking promotes carcinogenesis through multiple mechanisms, including oxidative stress, DNA damage, chronic inflammation, and immune dysregulation. In addition, it has been associated with accelerated progression of both chronic pancreatitis and pancreatic cancer, as well as worsening metabolic dysfunction [60]. In patients with MASLD, where systemic inflammation and metabolic stress are already present, tobacco exposure may exert synergistic effects, contributing to pancreatic injury, fibrosis, and tumour development.

Fatty pancreas, a metabolically related condition to MASLD, has also been associated with an increased risk of pancreatic cancer [61,62]. Meta-analyses and large cohort studies report approximately a two- to threefold higher risk among individuals with fatty pancreas compared with controls, potentially independent of traditional metabolic factors [63]. Proposed mechanisms include lipotoxicity, chronic inflammation, and a tumour-promoting microenvironment. However, no validated thresholds of pancreatic fat content or specific fat distribution patterns linked to cancer risk have been established, and residual confounding by metabolic comorbidities remains an important limitation. Consequently, the clinical utility of fatty pancreas as a pancreatic cancer risk marker or screening tool remains uncertain and requires prospective validation.

Overall, interpreting the association between MASLD and PC is challenging. The tumour itself may modify the metabolic phenotype before diagnosis, whether inducing new-onset or worsening diabetes or promoting weight loss, anorexia, alterations in lipid metabolism, potentially masking pre-existing MASLD. Selection and survival biases are also relevant given the high lethality and rapid course of pancreatic cancer, which may preclude timely liver assessment. In addition, CT, commonly used for diagnosis and staging, has limited sensitivity for mild steatosis and may lead to MASLD misclassification. Longitudinal studies incorporating pre-diagnostic metabolic data, medication exposure, prior imaging, and more sensitive liver phenotyping are therefore needed to clarify temporality and causality.

## 10. Impact of MASLD on Neo/Adjuvant Therapy in Pancreatic Cancer

Most patients with pancreatic cancer present with unresectable or metastatic disease at diagnosis, requiring systemic chemotherapy consideration [64]. In the context of MASLD, liver disease may influence tolerance, impact neoplastic response, and induce specific hepatic side effects.

These considerations are particularly relevant in the neoadjuvant setting. FOLFIRINOX, the preferred regimen for patients with resectable or borderline resectable disease, includes agents associated with an increased risk of liver injury. Fluoropyrimidines (5-fluorouracil and capecitabine) have been associated with radiographic hepatic steatosis in 30–47% of treated patients, likely through mitochondrial dysfunction and impaired fatty acid oxidation, which may further aggravate preexisting steatosis in patients with MASLD [65]. Irinotecan is associated with a 5-fold increased risk of chemotherapy-associated steatohepatitis and oxaliplatin has been linked to sinusoidal obstruction syndrome (SOS) [66]. Importantly, severe steatosis (≥30%) and steatohepatitis are associated with hepatic reserve impairment in this context, limiting treatment tolerance and adherence [67].

Beyond hepatotoxicity-related mechanisms, tumor metabolic reprogramming may also influence treatment response. Alterations in lipid homeostasis and increased de novo lipogenesis in pancreatic cancer cells contribute to tumorigenesis, neoplastic cells’ dissemination, and resistance to systemic therapy [68]. In this context, it is plausible that ectopic lipid accumulation and increased circulating FFAs may further modulate tumor metabolism and negatively affect treatment response. However, direct clinical evidence supporting this association remains limited and warrants further investigation.

Although current NCCN guidelines for PDAC do not provide MASLD-specific recommendations for treatment selection, available evidence supports careful baseline liver assessment and close hepatic monitoring during systemic therapy.

In this context, we recommend that patients with MASLD who are candidates for chemotherapy undergo a dedicated pre-treatment liver assessment, including exclusion of concomitant liver disease and fibrosis staging by transient elastography. If advanced fibrosis or cirrhosis is suspected, evaluation for clinically significant portal hypertension is warranted. In such cases, the expected oncological benefit of chemotherapy should be balanced against the risk of hepatic decompensation within a multidisciplinary setting, with close monitoring of liver function, portal hypertension-related complications, and treatment tolerance throughout therapy.

## 11. Pancreatic Surgery

Surgical resection remains the only curative option for PDAC, although fewer than 20% of patients are eligible for surgery [53]. Beyond malignancy, pancreatic surgery is also indicated for premalignant lesions, neuroendocrine tumours, AP local complications (i.e., necrosectomy) and CP patients with refractory pain or complications.

In this setting, the patient’s metabolic profile plays a key role in determining postoperative outcomes. Obesity and related disorders, such as MASLD, have been consistently associated with a higher incidence and severity of pancreatic complications, including postoperative pancreatic fistula (POPF), haemorrhage, and readmission rates [69]. Similarly, fatty pancreas has emerged as an important risk factor for adverse surgical outcomes, with imaging-based markers correlating with increased postoperative morbidity [70].

MASLD itself has also been associated with longer hospital stays and higher rates of pancreatic-specific complications following resection [71]. These risks appear to be compounded by the broader metabolic burden, including systemic inflammation and impaired tissue repair. In addition, in patients with advanced liver disease, the risk of hepatic decompensation after extrahepatic surgery is significantly increased. Factors such as sarcopenia or myosteatosis further worsen postoperative prognosis and are associated with poor overall survival and increased post- pancreaticoduodenectomy mortality [72].

Paradoxical hepatic steatosis has been described in malnourished patients, such as inanition or after metabolic surgery, due to impaired hepatic lipid metabolism. A similar phenomenon is observed following pancreatic resection, particularly pancreaticoduodenectomy, where MASLD prevalence ranges from 13% to over 30%, linked to pancreatic exocrine insufficiency and subsequent nutritional impairment [73,74]. Clinically, post-pancreatectomy MASLD has been associated with poorer survival and quality of life and prolonged hospitalisation [75,76]. Given these implications, recent predictive models incorporating clinical, imaging, and radiomics-based parameters have shown promise in identifying patients at higher risk of post-surgical resection MASLD [77].

Pancreatic enzyme replacement has been proposed as a preventive and therapeutic approach for post-PD MASLD, although current evidence remains inconclusive. Whilst some studies suggest improvements in hepatic steatosis and biochemical markers, controlled data have not demonstrated consistent benefit, and further data are needed [78,79].

Overall, these findings emphasise the central role of metabolic factors, including MASLD, in shaping surgical outcomes in pancreatic disease, especially pancreatic cancer. Preoperative optimisation through targeted nutritional and physical interventions may improve functional reserve and reduce postoperative morbidity, although evidence remains limited. Furthermore, structured postoperative nutritional surveillance is warranted, although further studies are needed to define effective strategies and clarify the impact of post-pancreatectomy MASLD on nutritional, hepatic, and oncologic outcomes.

## 12. Fatty Pancreas

Fatty pancreas (FP), formerly known as pancreatic steatosis, describes excessive fat accumulation within the pancreas, characterised by adipocyte infiltration of the intra- and/or interlobular spaces [80]. Epidemiological estimates remain highly heterogeneous, varying substantially according to population characteristics, diagnostic criteria, and imaging modalities; nevertheless, population-based studies report a 20–30% prevalence in the general population [81].

CMRF, particularly obesity, IR, and dyslipidemia, are the main triggers for FP development, as per MASLD, along with pancreatic-specific toxics, such as tobacco and alcohol. Amongst the shared pathophysiological pathways, IR plays a key role, since it promotes circulating FFAs that will accumulate both in the liver and in the pancreas, facilitating hepatic and pancreatic steatosis [82,83]. Consistent with this mechanism, histology-based studies have identified FP as a significant predictor of MASLD, an association that seems to be largely mediated by overall adiposity [84].

The bidirectional influence of FP and MASLD has been analysed in a recent meta-analysis comprising more than 60,000 subjects [85]. In this study, MASLD was associated with 6-fold odds of having FP, whilst the risk of MASLD was 9 times higher in patients with FP. Furthermore, the presence of pancreatic disease modifies MASLD natural history, since those patients with FP presented a higher risk of MASH and advanced fibrosis [86].

Importantly, these data should be interpreted with caution, since FP and MASLD detection and prevalence estimates rely on non-invasive imaging techniques accuracy and pre-defined thresholds. To date, US, CT, endoscopic ultrasound (EUS) and MRI have been tested for PS diagnosis (Table 2), although universally accepted diagnostic criteria and validated cut-off values are lacking [87,88,89,90,91]. Recently, an international consensus has provided the first standardized framework for the definition, diagnosis, classification, and management of fatty pancreas [80]. The consensus recognizes quantitative MRI techniques, particularly proton density fat fraction (PDFF), Dixon-based imaging, and MR spectroscopy, as the preferred methods for pancreatic fat quantification. Nevertheless, universally validated diagnostic thresholds and clinically meaningful outcome-based cut-offs remain unavailable.

In summary, although FP is consistently associated with MASLD and broader metabolic dysfunction, the underlying pathophysiological mechanisms remain incompletely understood. The relative contributions of metabolic, genetic, inflammatory, and obstructive factors to pancreatic fat accumulation are poorly defined, particularly in non-obese individuals, and much of the mechanistic evidence derives from animal models with limited human validation. Similarly, while pancreatic steatosis is linked to IR and T2D, it remains unclear whether fat accumulation directly drives endocrine and exocrine dysfunction or reflects a parallel manifestation of systemic metabolic stress, and whether pancreatic fat regression translates into functional recovery.

## 13. Considerations of MetALD (Metabolic Dysfunction and Alcohol-Associated Liver Disease) in Pancreatic Diseases

MetALD is a recently defined entity characterised by the presence of at least one CMRF and moderate alcohol intake, defined as 20–50 g/day in women and 30–60 g/day in men [92]. Although MetALD accounts for approximately 3% of all patients with SLD, misclassification as MASLD is common, mainly due to underreporting of alcohol consumption, suboptimal clinical assessment, and the limited availability of objective biomarkers [93,94].

In this regard, Staufer et al. reported that almost 30% of patients initially classified as presumed MASLD using questionnaires and non-specific alcohol biomarkers showed evidence of alcohol-related liver injury risk when ethyl glucuronide testing was performed [95]. These findings highlight the importance of systematic alcohol assessment in MASLD, particularly in patients at risk of pancreatic disease, given the direct pancreatic toxicity of alcohol.

The relationship between MetALD and pancreatic pathology is supported by overlapping pathophysiological mechanisms driven by the combined effects of metabolic dysfunction and alcohol exposure. The pancreas metabolizes ethanol through both oxidative and non-oxidative pathways, the latter generating fatty acid ethyl esters (FAEEs), which contribute to acinar cell injury [96]. Alcohol misuse remains one of the leading causes of pancreatic disease worldwide. Alcohol-induced pancreatic injury results from multiple interconnected mechanisms, including oxidative, mitochondrial and endoplasmic reticulum stress, intracellular calcium dysregulation, premature trypsinogen activation, inflammatory signalling, and extracellular matrix remodelling [97], which collectively promote acinar cell injury, necroinflammatory responses, and structural remodelling of the pancreatic parenchyma, thereby increasing the risk of acute and recurrent AP, as well as the progression to CP [98].

In patients with MetALD, the coexistence of alcohol exposure and metabolic dysfunction may further increase pancreatic susceptibility to injury, creating a biological milieu that favours persistent inflammation, fibrogenesis, and progression toward chronic pancreatitis.

In this context, the implementation of standardised diagnostic protocols in patients with MASLD, including a structured assessment and quantification of alcohol intake using biological biomarkers, is essential. Failure to accurately evaluate alcohol exposure may lead to misclassification and attribution bias, whereby pancreatic dysfunction is incorrectly ascribed to MASLD, overlooking a potential alcohol-related component. Moreover, unrecognised alcohol consumption may result in misdiagnosis, precluding identification of MetALD and limiting appropriate psychiatric evaluation for alcohol use disorder, as well as targeted assessment and management of pancreatic complications such as exocrine insufficiency.

## 14. Nutritional Intricacies in Patients with Liver and Major Pancreatic Disorders

Liver and pancreatic diseases includes systemic inflammation, increased catabolism, reduced oral intake and malabsorption, which may lead to nutritional impairment. Early nutritional screening, structured body composition assessment, and individualised dietary and lifestyle interventions are therefore essential, particularly in patients with coexisting liver and pancreatic disorders.

In advanced liver disease, malnutrition results from reduced oral intake due to dysgeusia and early satiety, along with malabsorption secondary to intestinal edema, small intestinal bacterial overgrowth, and impaired bile acid metabolism. In parallel, cirrhosis induces an accelerated starvation state characterised by depleted glycogen stores, increased gluconeogenesis, lipolysis and proteolysis [99].

Nutritional risk screening in patients with advanced liver disease should be performed using liver-specific tools, such as the Royal Free Hospital–Nutritional Prioritising Tool [100]. Malnutrition diagnosis and stratification should follow standardised criteria, such as the Global Leadership Initiative on Malnutrition (GLIM) framework [101], in which the mere presence of sarcopenia indicates severe malnutrition. Given the high prevalence of sarcopenia in cirrhosis, affecting approximately 30–40% of patients overall and up to 60% of those with decompensated disease [102], systematic body composition assessment becomes mandatory.

Although alcohol-related liver disease remains the main etiology associated with nutritional impairment, MASLD poses a distinct challenge, as excess adiposity may mask nutritional deficiencies and muscle abnormalities, such as sarcopenia, sarcopenic obesity and myosteatosis, which are associated with impaired muscle quality and adverse outcomes [103]. This is particularly relevant as MASLD treatment relies on weight loss (≥7–10% in overweight or ≥3–5% in lean patients) to improve liver-related outcomes [104]. Importantly, this target should be achieved without compromising lean mass, especially in older adults, patients with cirrhosis or sarcopenic obesity, and those receiving incretin-based therapies, whose effects on muscle mass and function remain insufficiently characterised [105].

The Mediterranean diet is the dietary pattern with the strongest evidence in MASLD, given its beneficial effects on both liver-related and cardiovascular outcomes [106]. Within this framework, emerging evidence suggests that prioritising plant-based protein sources over animal-derived proteins may confer additional metabolic and gut–liver axis benefits in this population [107].

In patients with advanced liver disease, nutritional therapy should prioritise adequate energy and protein intake, avoidance of prolonged fasting, and preservation of muscle mass and function (Table 3). Patients should generally receive 30–35 kcal/kg/day and 1.2–1.5 g/kg/day of protein, increasing protein intake when sarcopenia or acute decompensation occurs. Meals should be distributed throughout the day, with emphasis on a protein-containing breakfast and a late-evening snack combining complex carbohydrates and protein to reduce nocturnal catabolism [99].

On the other hand, the nutritional burden and management of pancreatic disorders vary according to disease type and severity (Table 4). In acute pancreatitis, nutritional risk is mainly driven by systemic inflammation, organ failure and fasting duration, and is particularly relevant in severe disease, pre-existing malnutrition, alcohol-related AP and concomitant obesity. Malnutrition has been reported in approximately 14% of AP patients, whereas every severe AP should be considered a high nutritional-risk condition because of its marked inflammatory and catabolic burden [108].

Generally speaking, nutritional management in AP has shifted from prolonged fasting towards early refeeding. In mild AP, oral intake should be resumed as soon as clinically tolerated, usually with a low-fat, soft diet. If oral intake is not tolerated or remains insufficient, enteral nutrition should be preferred over parenteral nutrition to preserve gut barrier function and reduce infectious complications. In severe AP receiving enteral nutrition, intra-abdominal pressure should be monitored given the risk of abdominal compartment syndrome. In AP with local complications (i.e., WON requiring necrosectomy), oral feeding may be restarted within 24 h if the patient is clinically stable [109].

In chronic pancreatitis, malnutrition and sarcopenia have been reported in 40–60% and 40% of patients, respectively [110]. Nutritional impairment in CP is driven by abdominal pain, reduced intake, alcohol and tobacco use, increased energy expenditure and PEI. Routine fat restriction should be avoided, as it may worsen energy deficits and impair nutritional recovery. Pancreatic enzyme replacement therapy (PERT) generally starts with at least 25,000–50,000 PhU with main meals and half this dose with snacks and should be optimised according to meal size, fat content, symptoms, and nutritional response. In malnourished patients, high-energy, high-protein foods divided into five to six small meals per day are recommended [109,111].

Pancreatic cancer is characterised by early and profound nutritional deterioration, driven by systemic inflammation, tumour-related metabolic derangements, anorexia, or pancreatic exocrine insufficiency. Cachexia and sarcopenia, conditions associated with poorer treatment tolerance, quality of life and survival, affect approximately 70 and 50% of patients, respectively [112,113]. In PC, energy intake should target 25–30 kcal/kg/day, while protein ingestion should reach 1.2–1.5 g/kg/day, especially in patients with malnutrition or sarcopenia. Importantly, PERT should be promptly prescribed early when PEI is suspected to improve symptom control and nutritional status.

In both liver and pancreatic disorders, resistance and aerobic exercise should complement nutritional intervention to preserve muscle mass, strength and physical function. Early detection and management of malnutrition and sarcopenia, together with tailored lifestyle interventions, are essential to improve patient outcomes.

From a nutritional and clinical perspective, managing patients with MASLD and concomitant pancreatic disease, particularly those with obesity, acute inflammation, or PEI, requires balancing weight reduction with preservation of lean mass and nutritional status. We recommend standardised nutritional and body-composition assessment using GLIM criteria, bioelectrical impedance analysis, and L3-CT measurements, together with supervised aerobic and resistance exercise, an individualised high-protein diet, and PERT and micronutrient supplementation when indicated. Weight-lowering pharmacotherapy should be used cautiously, or deferred, in patients with malnutrition or sarcopenia until nutritional status is stabilised. Emerging combinations with lean-mass–preserving agents may offer future alternatives, although current evidence remains preliminary [114]. Finally, high-risk patients should undergo close follow-up, with body-composition reassessment every 3–6 months by BIA and annually by L3-CT when clinically available.

## 15. MASLD Association with Less Frequent Pancreatic Disorders

### 15.1. Autoimmune Pancreatitis

Autoimmune pancreatitis (AIP) is a chronic pancreatic inflammatory disorder characterised by lymphoplasmacytic infiltration and steroid responsiveness. It typically presents with obstructive jaundice, with or without a pancreatic mass, and is frequently diagnosed in the context of suspected malignancy or CP. AIP is rare (1/100,000 annually in Western countries) and comprises two subtypes: type 1 (AIP-1), the pancreatic manifestation of IgG4-related disease, and type 2 (AIP-2), a pancreas-specific, non-IgG4-associated entity [115].

IgG4-related liver disease, primary sclerosing cholangitis, and autoimmune hepatitis (AIH) are the most frequent hepatic disorders related to AIP [116]. Although MASLD is now recognised as a chronic inflammatory state and its association with systemic autoimmune diseases (e.g., psoriasis, inflammatory bowel disease) is established [117,118,119], the relationship of MASLD with AIP is reduced to a single case reported by Matsumoto et al., who described an IgG4-related AIP with IgG4-related liver disease and biopsy-proven MASH [120].

Importantly, MASLD can coexist with hepatic autoimmune diseases, and its diagnosis may be challenging due to overlapping clinical, biochemical, and histologic features. This overlap may result in MASLD underdiagnosis in an autoimmune context, highlighting the need for heightened suspicion in patients with immune-mediated disorders and/or metabolic risk factors [121].

### 15.2. Cystic Fibrosis

Cystic fibrosis (CF) is the most common autosomal recessive disease in Caucasians, caused by CFTR mutations that impair chloride transport and produce viscous secretions [122].

CF hepatobiliary involvement (CFHBI) occurs in 30–40% of CF patients and represents the third leading cause of CF-related mortality. CFHBI ranges from asymptomatic enzyme elevations to cholelithiasis, liver fibrosis and even cirrhosis [123], with hepatic steatosis being the most common manifestation, affecting 20–60% of patients [124]. Once thought to result from malnutrition, recent studies linked steatosis with preserved nutritional status and normal pulmonary function. Notably, CF-related steatosis shows no consistent association with traditional metabolic risk factors, alcohol use, or CF-related diabetes [125]. Moreover, specific CF medications, such as CFTR modulators (lumacaftor/ivacaftor), have been shown to reduce liver fat on MRI-PDFF [126]. Together, these findings suggest an intrinsic predisposition to liver steatosis in CF, warranting further investigation into its relationship with MASLD.

### 15.3. Pancreatic Cysts

Pancreatic cystic lesions include serous cystadenoma, intraductal papillary mucinous neoplasm (IPMN), mucinous cystic neoplasm (MCN), solid pseudopapillary neoplasm, and cystic neuroendocrine tumour. Serous cystadenomas are benign, whereas mucinous lesions (IPMN, MCN), solid pseudopapillary neoplasms, and cystic neuroendocrine tumours carry variable malignant potential [127].

Evidence linking pancreatic cysts and MASLD is preliminary. In a case–control study, Sbeit et al. observed higher odds of serous and mucinous cystadenomas and of branch-duct/mixed-type IPMN in MASLD patients, independent of the presence of fatty pancreas [128]. In another cohort study, Tanka et al. reported an association of MetS with a steeper age-related rise in IPMN prevalence, suggesting a broader metabolic milieu that may favour cyst initiation or progression [129]. Overall, converging direct and indirect data suggest an association between pancreatic cysts and MASLD, modulated by metabolic risk. Prospective and mechanistic studies are needed to determine causality, define MASLD’s role in cyst progression, and clarify any impact on malignant transformation.

## 16. Future Directions and Research Priorities

This review highlights the clinical relevance of pancreatic involvement in MASLD and underscores several knowledge gaps that warrant further investigation.

Future research should prioritise a deeper understanding of the pathophysiological links between MASLD and pancreatic disease. Establishing whether MASLD has a causal and independent role in pancreatic disorders will require addressing confounding, reverse causation, and time-dependent exposures. Prospective cohorts should incorporate standardised assessment of liver steatosis and fibrosis, pancreatic fat and function, body composition, alcohol and tobacco use, diet, socioeconomic determinants and longitudinal metabolic control, together with pancreatic outcomes. These should be complemented by causal-inference approaches, including Mendelian randomisation and target-trial emulation, together with sibling, negative-control, mechanistic, and interventional analyses, to distinguish the effect of MASLD itself from that of its associated cardiometabolic burden [130,131,132].

The clinical implications of MASLD in pancreatic disorders also deserve dedicated attention. The role of MASLD in pancreatic exocrine and endocrine dysfunction, especially in the absence of CP, remains poorly defined. In addition, the integration of hepatic and metabolic parameters into prognostic models, particularly in acute pancreatitis, should be explored and validated, as they are key determinants of AP severity.

Nutrition-focused interventions in patients with MASLD and pancreatic disease should be prospectively evaluated, along with the effects of weight-loss strategies on lean mass, nutritional and functional status in this population. In addition, standardised assessment of malnutrition and body composition should be incorporated into routine clinical practice to guide personalised care.

Pancreatic steatosis is an increasingly recognised entity with potential metabolic and local consequences; however, standardisation of its diagnosis is still needed. This includes validating non-invasive imaging techniques against histology and establishing widely accepted diagnostic thresholds. Until clinically validated outcome-based thresholds become available, future studies should adopt standardized MRI-PDFF acquisition and reporting protocols, with appropriate adjustment for major metabolic confounders.

From an oncological perspective, further studies are required to better define the impact of MASLD on tumour biology, treatment response, and clinical outcomes. These insights may inform future risk stratification models and screening strategies in selected high-risk populations. In addition, the influence of MASLD on tolerance to systemic therapies and postoperative outcomes highlights the need to incorporate metabolic assessment into decision-making pathways.

Finally, while metabolic therapies are becoming central in MetS and MASLD management [133], their impact on pancreatic disease is poorly explored [134]. The effects of weight loss interventions, pharmacological treatments such as glucagon-like peptide-1 receptor agonists, and bariatric surgery on pancreatic steatosis and related outcomes should be investigated. Future longitudinal and interventional studies are needed to determine the clinical consequences of pancreatic fat regression, including its effects on pancreatic endocrine and exocrine function and its potential impact on pancreatic cancer risk. In parallel, improved assessment of alcohol exposure and clearer differentiation between MASLD and MetALD are essential to reduce misclassification and optimise patient care.

## 17. Conclusions

The association between MASLD and pancreatic diseases is supported by a growing body of evidence, highlighting a robust and clinically relevant link. Beyond shared CMRF, emerging data suggest that MASLD may actively contribute to pancreatic dysfunction and disease progression, reinforcing the concept of a bidirectional liver–pancreas axis.

These findings underscore the importance of considering MASLD as a systemic condition with potential implications beyond the liver, including its role in pancreatic disorders. Recognition of this association is essential for improving risk stratification, diagnostic evaluation, and clinical management. Importantly, nutritional and body composition assessment is essential in patients with liver and pancreatic disorders, given the high prevalence and major clinical impact of malnutrition and sarcopenia in this population.

However, significant knowledge gaps remain. Further studies are needed to elucidate causal pathways and to better define the role of MASLD in pancreatic disease, particularly to clarify causal mechanisms and define its role in disease progression and outcomes.

Overall, advancing this field will require a multidisciplinary approach integrating hepatology, pancreatology, oncology, and metabolic medicine, with the aim of improving early recognition, personalised management, and patient outcomes.

## Figures and Tables

**Table 1:** MASLD-associated comorbidities.

**Cardiometabolic Risk Factors**	**Neoplasms**
Obesity	Hepatocellular carcinoma
Type 2 diabetes	Cholangiocarcinoma
Dyslipidemia	Esophageal cancer
Hypertension	Gastric cancer
**Cardiovascular Diseases**	Colorectal cancer
Coronary artery disease	Thyroid cancer
Atherosclerosis	Breast cancer
Heart failure	Genitourinary cancer
Atrial fibrillation	Lung cancer
Valvular heart disease	Gallbladder cancer
Stroke	**Immune-Related Conditions**
Chronic kidney disease (CKD)	Dermatological-based disease (psoriasis, hidradenitis)
**Endocrine Organ Dysfunction**	Rheumatological diseases (rheumatoid arthritis, lupus, psoriatic arthritis…)
Hypothyroidism/Hashimoto’s thyroiditis	Digestive system diseases (celiac disease, inflammatory bowel disease, AIH, PBC, PSC)
Polycystic ovary syndrome (PCOS)	**Gut dysbiosis**
Male hypogonadism	**Others**
Type 1 diabetes	Hyperuricemia
**Pancreatic Diseases**	Obstructive Sleep Apnea Syndrome (OSAS)
Acute pancreatitis	Cognitive impairment/Dementia
Chronic pancreatitis	Urolithiasis
Pancreatic cancer	Gallstone disease
Fatty pancreas	
Cystic fibrosis, autoimmune pancreatitis	

**Table 2:** Imaging techniques for fatty pancreas diagnosis and stratification.

	Description	Classification/Cut-off Points	Pros/Cons
**Abdominal Ultrasound (US)** [83]	Compare pancreatic with kidney and retroperitoneal fat echogenicity.	Grade 0: Similar echogenicity to the kidney. Grade 1: Pancreatic echogenicity mildly increased compared to the kidney. Grade 2: Markedly higher echogenicity than the kidney but lower than retroperitoneal fat. Grade 3: Higher echogenicity than retroperitoneal fat.	Quick, accessible, non-invasive. Low sensitivity for mild steatosis. Operator-dependent
**Computed Tomography (CT)** [84]	Detects fat infiltration based on Hounsfield unit density.	Grade 0: Normal Grade 1: Fatty replacement <25% of the pancreatic region. Grade 2: 25–50% fatty replacement Grade 3: 50–75% fatty replacement Grade 4: >75% fatty replacement	Useful for quantifying regional fat infiltration. Less sensitive to focal steatosis.
**Endoscopic Ultrasound (EUS)** [76,85]	Compare pancreatic echogenicity to the spleen.	Grade 0: Normal. Grade 1/2: Hyperechoic pancreas compared to spleen or kidney, partial obscuration of anatomical landmarks and MPD Grade 3: Severe hyperechogenicity, adjacent structures and MPD not visible.	Highly sensitive. Diagnostic criteria were not standardised. Operator-dependent.
**Magnetic Resonance Imaging (MRI)** [86]	Quantifies fat proton density fat fraction.	PDFF > 6.2–6.4% is considered indicative of pancreatic steatosis.	Most accurate and allows quantification. Expensive. Low availability.

**Table 3:** Main nutritional recommendations in advanced liver disease.

	Main Recommendation
**Malnutrition screening**	Perform routine nutritional screening. High risk: BMI < 18.5 kg/m^2^ or Child–Pugh C. Use validated tools (RFH-NPT and GLIM criteria)
**Nutritional assessment**	Detailed assessment including dietary intake, weight changes, meal pattern, barriers to eating, and sodium intake.
**Sarcopenia assessment**	Assess muscle mass and function. Preferred CT-L3; alternatives include anthropometry, DEXA or BIA. Handgrip strength for function.
**Meal distribution**	Small, frequent meals: 3 meals + 3 snacks/day. Include breakfast and a late-evening snack to avoid prolonged fasting.
**Energy intake**	30–35 kcal/kg/day, preferably calculated using dry body weight in patients with ascites/edema.
**Protein intake**	1.2–1.5 g/kg/day increasing >1.5 if sarcopenia. Avoid protein restriction. Consider vegetable or dairy protein if meat protein is poorly tolerated.
**Micronutrients**	Screen and correct deficiencies, especially vitamin D, fat-soluble vitamins, thiamine/B vitamins, zinc, selenium, magnesium, calcium and iron.
**Nutritional supplements**	Use oral nutritional supplements; consider enteral nutrition if intake remains insufficient. Parenteral nutrition only if oral/enteral feeding is not feasible.
**Exercise**	Combine nutrition with individualized resistance and aerobic exercise to preserve muscle mass and function.

**Table 4:** Main nutritional recommendations in AP and CP.

Area	AP Recommendation	CP Recommendation
**Nutritional risk**	Consider AP a moderate-to-high nutritional-risk condition, especially in severe AP, malnutrition, alcohol-related disease, or obesity.	Consider CP a high-risk condition for malnutrition, sarcopenia, micronutrient deficiencies, and bone disease.
**Nutritional assessment and screening**	Screen using validated tools such as NRS-2002	Assess symptoms, dietary intake, weight change, functional status and body composition. Screen for micro- and macronutrient deficiencies at least annually, including A, D, E, K vitamins; vitamin B12, folate, thiamine; magnesium, iron, selenium, and zinc
**Diet pattern**	Use a low-fat, soft oral diet when restarting feeding, with an early, swift transition to the conventional diet.	Balanced diet without unnecessary dietary restriction, avoiding very high-fibre diets. Restrict fat only if steatorrhea persists despite optimized enzyme therapy.
**Oral intolerance** **Enteral nutrition**	Enteral nutrition preferred. Early initiation (first 24–72 h of admission) using a standard polymeric formula. Semi-elemental formulas may be considered if malabsorption or intolerance occurs. Nasogastric feeding as first option.	Consider enteral nutrition when oral nutrition and supplements are insufficient. Jejunal access preferred.
**Parenteral nutrition**	For patients with enteral nutrition intolerance, contraindications or ineffectiveness. Parenteral glutamine may be supplemented at 0.20 g/kg/day.	Reserve for gastric outlet obstruction, complex fistulating disease, enteral feeding intolerance, or inability to meet requirements enterally.
**Pancreatic enzymes**	Do not use routinely; only in obvious PEI.	Initiate when steatorrhea, weight loss, malabsorption, or malnutrition.

## Data Availability

The original contributions presented in this study are included in the article. Further inquiries can be directed to the corresponding author.

## References

[1] Younossi Z.M., Kalligeros M., Henry L. (2025). Epidemiology of metabolic dysfunction-associated steatotic liver disease. Clin. Mol. Hepatol..

[2] Byrne D., Targher G. (2015). NAFLD: A multisystem disease. J. Hepatol..

[3] Targher G., Byrne C.D., Tilg H. (2024). MASLD: A systemic metabolic disorder with cardiovascular and malignant complications. Gut.

[4] van der Zijl N.J., Goossens G.H., Moors C.C., van Raalte D.H., Muskiet M.H., Pouwels P.J., Blaak E.E., Diamant M. (2011). Ectopic fat storage in the pancreas, liver, and abdominal fat depots: Impact on β-cell function in individuals with impaired glucose metabolism. J. Clin. Endocrinol. Metabol..

[5] Rinella M.E., Lazarus J.V., Ratziu V., Francque S.M., Sanyal A.J., Kanwal F., Romero D., Abdelmalek M.F., Anstee Q.M., Arab J.P. (2023). A multisociety Delphi consensus statement on new fatty liver disease nomenclature. J. Hepatol..

[6] Hagström H., Shang Y., Hegmar H., Nasr P. (2024). Natural history and progression of metabolic dysfunction-associated steatotic liver disease. Lancet Gastroenterol. Hepatol..

[7] Rafiq N., Bai C., Fang Y., Srishord M., McCullough A., Gramlich T., Younossi Z.M. (2009). Longterm follow-up of patients with nonalcoholic fatty liver. Clin. Gastroenterol. Hepatol..

[8] Huang D.Q., El-Serag H.B., Loomba R. (2021). Global epidemiology of NAFLD-related HCC: Trends, predictions, risk factors and prevention. Nat. Rev. Gastroenterol. Hepatol..

[9] Rivera-Esteban J., Muñoz-Martínez S., Higuera M., Sena E., Bermúdez-Ramos M., Bañares J., Martínez-Gomez M., Cusidó M.S., Jiménez-Masip A., Francque S.M. (2024). Phenotypes of Metabolic Dysfunction-Associated Steatotic Liver Disease-Associated Hepatocellular Carcinoma. Clin. Gastroenterol. Hepatol..

[10] Zhou B.G., Jiang X., She Q., Ding Y.B. (2024). Association of MASLD with the risk of extrahepatic cancers: A systematic review and meta-analysis of 18 cohort studies. Eur. J. Clin. Investig..

[11] Bansal S.K., Bansal M.B. (2024). Pathogenesis of MASLD and MASH—Role of insulin resistance and lipotoxicity. Aliment. Pharmacol. Ther..

[12] Bessone F., Razori M.V., Roma M.G. (2019). Molecular pathways of nonalcoholic fatty liver disease development and progression. Cell Mol. Life Sci..

[13] Pu S., Zhao B., Jiang Y., Cui X. (2025). Hypothyroidism/subclinical hypothyroidism and metabolic dysfunction-associated steatotic liver disease: Advances in mechanism and treatment. Lipids Health Dis..

[14] Manzano-Nunez R., Santana-Dominguez M., Rivera-Esteban J., Sabiote C., Sena E., Bañares J., Tacke F., Pericàs J.M. (2023). Non-Alcoholic Fatty Liver Disease in Patients with Polycystic Ovary Syndrome: A Systematic Review, Meta-Analysis, and Meta-Regression. J. Clin. Med..

[15] Vujasinovic M., Ebrahimi F., Roelstraete B., Bergman D., Sun J., Sadr-Azodi O., Löhr J.-M., Ludvigsson J.F. (2025). Metabolic Dysfunction-Associated Steatotic Liver Disease and Pancreatic Disease-A Population-Based Nationwide Cohort and Sibling-Controlled Study. United Eur. Gastroenterol. J..

[16] Li T., Qin C., Zhao B., Li Z., Zhao Y., Lin C., Wang W. (2024). Global and regional burden of pancreatitis: Epidemiological trends, risk factors, and projections to 2050 from the global burden of disease study 2021. BMC Gastroenterol..

[17] Li T., Lin C., Wang W. (2025). Global, regional, and national burden of pancreatic cancer from 1990 to 2021, its attributable risk factors, and projections to 2050: A systematic analysis of the global burden of disease study 2021. BMC Cancer.

[18] Zhou M., Lv J., Chen X., Shi Y., Chao G., Zhang S. (2025). From gut to liver: Exploring the crosstalk between gut-liver axis and oxidative stress in metabolic dysfunction-associated steatotic liver disease. Ann. Hepatol..

[19] Lee S.H., Park S.Y., Choi C.S. (2022). Insulin Resistance: From Mechanisms to Therapeutic Strategies. Diabetes Metab. J..

[20] Chang M., Chen W., Xia R., Peng Y., Niu P., Fan H. (2023). Pancreatic Stellate Cells and the Targeted Therapeutic Strategies in Chronic Pancreatitis. Molecules.

[21] Valente R., Coppola A., Scandavini C.M., Halimi A., Magnusson A., Lauro A., Sotirova I., Arnelo U., Franklin O. (2024). Interactions between the Exocrine and the Endocrine Pancreas. J. Clin. Med..

[22] Stefan N., Schick F., Birkenfeld A.L., Häring H.U., White M.F. (2023). The role of hepatokines in NAFLD. Cell Metab..

[23] Barros D.R., Hegele R.A. (2025). Fibroblast growth factor 21: Update on genetics and molecular biology. Curr. Opin. Lipidol..

[24] Albillos A., de Gottardi A., Rescigno M. (2020). The gut-liver axis in liver disease: Pathophysiological basis for therapy. J. Hepatol..

[25] Pushalkar S., Hundeyin M., Daley D., Zambirinis C.P., Kurz E., Mishra A., Mohan N., Aykut B., Usyk M., Torres L.E. (2018). The Pancreatic Cancer Microbiome Promotes Oncogenesis by Induction of Innate and Adaptive Immune Suppression. Cancer Discov..

[26] Petersen O.H., Gerasimenko J.V., Gerasimenko O.V., Gryshchenko O., Peng S. (2021). The roles of calcium and ATP in the physiology and pathology of the exocrine pancreas. Physiol. Rev..

[27] Petersen O.H. (2023). The 2022 George E Palade Medal Lecture: Toxic Ca^2+^ signals in acinar, stellate and endogenous immune cells are important drivers of acute pancreatitis. Pancreatology.

[28] Navina S., Acharya C., DeLany J.P., Orlichenko L.S., Baty C.J., Shiva S.S., Durgampudi C., Karlsson J.M., Lee K., Bae K.T. (2011). Lipotoxicity causes multisystem organ failure and exacerbates acute pancreatitis in obesity. Sci. Transl. Med..

[29] Wu J., Zhang L., Shi J., He R., Yang W., Habtezion A., Niu N., Lu P., Xue J. (2020). Macrophage phenotypic switch orchestrates the inflammation and repair/regeneration following acute pancreatitis injury. EBioMedicine.

[30] Liu B., Zhang X., Li J., Sun Z., Lin C., Ning C., Hong X., Zhu S., Shen D., Chen L. (2025). The Global, Regional, and National Burden of Pancreatitis in 204 Countries and Territories, 1990–2021: A Systematic Analysis for the Global Burden of Disease Study 2021. Dig. Dis. Sci..

[31] Boxhoorn L., Voermans R.P., Bouwense S.A., Bruno M.J., Verdonk R.C., Boermeester M.A., van Santvoort H.C., Besselink M.G. (2020). Acute pancreatitis. Lancet.

[32] van Dijk S.M., Hallensleben N.D.L., van Santvoort H.C., Fockens P., van Goor H., Bruno M.J., Besselink M.G., Dutch Pancreatitis Study Group (2017). Acute pancreatitis: Recent advances through randomised trials. Gut.

[33] Roberts S.E., Morrison-Rees S., John A., Williams J.G., Brown T.H., Samuel D.G. (2017). The incidence and aetiology of acute pancreatitis across Europe. Pancreatology.

[34] Váncsa S., Sipos Z., Váradi A., Nagy R., Ocskay K., Juhász F.M., Márta K., Teutsch B., Mikó A., Hegyi P.J. (2023). Metabolic-associated fatty liver disease is associated with acute pancreatitis with more severe course: Post hoc analysis of a prospectively collected international registry. United Eur. Gastroenterol. J..

[35] Zhang Y., Zhang J., Yu D., Tu F., Liu J., Han B., Li B., Yuan Y., Chen C., Zhou M. (2025). Association between metabolic dysfunction associated steatotic liver disease and gallstones in the US population using propensity score matching. Sci. Rep..

[36] Gu S., Hu S., Wang S., Qi C., Shi C., Fan G. (2023). Bidirectional association between NAFLD and gallstone disease: A systematic review and meta-analysis of observational studies. Expert Rev. Gastroenterol. Hepatol..

[37] Szentesi A., Párniczky A., Vincze Á., Bajor J., Gódi S., Sarlós P., Gede N., Izbéki F., Halász A., Márta K. (2019). Multiple Hits in Acute Pancreatitis: Components of Metabolic Syndrome Synergize Each Other’s Deteriorating Effects. Front. Physiol..

[38] Sbeit W., Khoury T. (2021). Fatty Pancreas Represents a Risk Factor for Acute Pancreatitis: A Pilot Study. Pancreas.

[39] Hoferica J., Borbély R.Z., Aghdam A.N., Szalai E.Á., Zolcsák Á., Veres D.S., Hagymási K., Erőss B., Hegyi P., Bánovčin P. (2024). Chronic liver disease is an important risk factor for worse outcomes in acute pancreatitis: A systematic review and meta-analysis. Sci. Rep..

[40] Zhang Q., Liu P., Xu X., Yang Y., Xiong Y. (2026). Clinical Outcomes of Acute Pancreatitis in Patients with Metabolic Dysfunction-Associated Fatty Liver Disease (MAFLD): A Systematic Review and Meta-Analysis. Dig. Dis. Sci..

[41] Olesen S.S., Mortensen L.H., Zinck E., Becker U., Drewes A.M., Nøjgaard C., Novovic S., Yadav D., Tolstrup J.S. (2021). Time trends in incidence and prevalence of chronic pancreatitis: A 25-year population-based nationwide study. United Eur. Gastroenterol. J..

[42] Dominguez-Munoz J.E., Drewes A.M., Lindkvist B., Ewald N., Czakó L., Rosendahl J., Löhr J.M., Löhr M., Dominguez-Munoz J.E., Besselink M. (2018). Recommendations from the United European Gastroenterology evidence-based guidelines for the diagnosis and therapy of chronic pancreatitis. Pancreatology.

[43] Gandhi S., de la Fuente J., Murad M.H., Majumder S. (2022). Chronic Pancreatitis Is a Risk Factor for Pancreatic Cancer, and Incidence Increases With Duration of Disease: A Systematic Review and Meta-analysis. Clin. Transl. Gastroenterol..

[44] Cai Q.Y., Tan K., Zhang X.L., Han X., Pan J.P., Huang Z.Y., Tang C.W., Li J. (2023). Incidence, prevalence, and comorbidities of chronic pancreatitis: A 7-year population-based study. World J. Gastroenterol..

[45] Márta K., Hegyi P. (2019). Uncommon appearance of concurrent liver cirrhosis and chronic pancreatitis: The alcohol metabolism theory. Dig. Liver Dis..

[46] Saluja S.S., Varshney V.K., Kumar A., Sugumaran K., Aravinda P.S., Mishra P.K. (2022). Histopathological Changes in Liver of Patients With Chronic Pancreatitis Requiring Surgical Intervention and Their Clinical Significance. Pancreas.

[47] Dominguez-Muñoz J.E., Vujasinovic M., de la Iglesia D., Cahen D., Capurso G., Gubergrits N., Hegyi P., Hungin P., Ockenga J., Paiella S. (2025). European guidelines for the diagnosis and treatment of pancreatic exocrine insufficiency: UEG, EPC, EDS, ESPEN, ESPGHAN, ESDO, and ESPCG evidence-based recommendations. United Eur. Gastroenterol. J..

[48] Ewald N., Bretzel R.G. (2013). Diabetes mellitus secondary to pancreatic diseases (Type 3c)-are we neglecting an important disease?. Eur. J. Intern. Med..

[49] Cusi K., Abdelmalek M.F., Apovian C.M., Balapattabi K., Bannuru R.R., Barb D., Bardsley J.K., Beverly E.A., Corbin K.D., ElSayed N.A. (2025). Metabolic Dysfunction–Associated Steatotic Liver Disease (MASLD) in People with Diabetes: The Need for Screening and Early Intervention. A Consensus Report of the American Diabetes Association. Diabetes Care.

[50] Boga S., Koksal A.R., Sen İ., Kurul Yeniay M., Yilmaz Ozguven M.B., Serin E., Erturk S.M., Alkim H., Alkim C. (2020). Liver and pancreas: ‘Castor and Pollux’ regarding the relationship between hepatic steatosis and pancreas exocrine insufficiency. Pancreatology.

[51] Wen W.H., Chen H.L., Chang M.H., Ni Y.H., Shih H.H., Lai H.S., Hsu W.M. (2005). Fecal elastase 1, serum amylase and lipase levels in children with cholestasis. Pancreatology.

[52] Sung H., Ferlay J., Siegel R.L., Laversanne M., Soerjomataram I., Jemal A., Bray F. (2021). Global Cancer Statistics 2020: GLOBOCAN Estimates of Incidence and Mortality Worldwide for 36 Cancers in 185 Countries. CA Cancer J. Clin..

[53] Mizrahi J.D., Surana R., Valle J.W., Shroff R.T. (2020). Pancreatic cancer. Lancet.

[54] Mantovani A., Petracca G., Beatrice G., Csermely A., Tilg H., Byrne C.D., Targher G. (2022). Non-alcoholic fatty liver disease and increased risk of incident extrahepatic cancers: A meta-analysis of observational cohort studies. Gut.

[55] Allen A.M., Hicks S.B., Mara K.C., Larson J.J., Therneau T.M. (2019). The risk of incident extrahepatic cancers is higher in non-alcoholic fatty liver disease than obesity—A longitudinal cohort study. J. Hepatol..

[56] Park J.H., Hong J.Y., Han K., Kang W., Park J.K. (2022). Increased risk of pancreatic cancer in individuals with non-alcoholic fatty liver disease. Sci. Rep..

[57] Souza M., Diaz I., Barchetta I., Mantovani A. (2024). Gastrointestinal cancers in lean individuals with non-alcoholic fatty liver disease: A systematic review and meta-analysis. Liver Int..

[58] Yu Q., Song H., Shi X.Y., Zhu L., Liang Y., Gong R.N., Dong X.W., Liu S.L., Wang H.Z., Wang Y.L. (2026). Metabolic dysfunction-associated steatotic liver disease accelerates pancreatic cancer progression and metastasis via the macrophage migration inhibitory factor-CD44 axis. Signal Transduct. Target. Ther..

[59] Marti-Aguado D., Clemente-Sanchez A., Bataller R. (2022). Cigarette smoking and liver diseases. J. Hepatol..

[60] Yadav D., Hawes R.H., Brand R.E., Anderson M.A., Money M.E., Banks P.A., Bishop M.D., Baillie J., Sherman S., DiSario J. (2009). Alcohol consumption, cigarette smoking, and the risk of recurrent acute and chronic pancreatitis. Arch. Intern. Med..

[61] Lipp M., Tarján D., Lee J., Zolcsák Á., Szalai E., Teutsch B., Faluhelyi N., Erőss B., Hegyi P., Mikó A. (2023). Fatty Pancreas Is a Risk Factor for Pancreatic Cancer: A Systematic Review and Meta-Analysis of 2956 Patients. Cancers.

[62] Vlăduț C., Steiner C., Löhr M., Gökçe D.T., Maisonneuve P., Hank T., Öhlund D., Sund M., Hoogenboom S.A. (2025). High prevalence of pancreatic steatosis in pancreatic cancer patients: A meta-analysis and systematic review. Pancreatology.

[63] Christakoudi S., Tsilidis K.K., Gunter M.J., Riboli E. (2025). Prospective Associations of Body Composition and Body Shape With the Risk of Developing Pancreatic Cancer in the UK Biobank Cohort. Cancer Med..

[64] McGuigan A., Kelly P., Turkington R.C., Jones C., Coleman H.G., McCain R.S. (2018). Pancreatic cancer: A review of clinical diagnosis, epidemiology, treatment and outcomes. World J. Gastroenterol..

[65] Chun Y.S., Laurent A., Maru D., Vauthey J.N. (2009). Management of chemotherapy-associated hepatotoxicity in colorectal liver metastases. Lancet Oncol..

[66] Vauthey J.N., Pawlik T.M., Ribero D., Wu T.T., Zorzi D., Hoff P.M., Xiong H.Q., Eng C., Lauwers G.Y., Mino-Kenudson M. (2006). Chemotherapy regimen predicts steatohepatitis and an increase in 90-day mortality after surgery for hepatic colorectal metastases. J. Clin. Oncol..

[67] Truant S., Baillet C., Gnemmi V., Fulbert M., Turpin A., Dardenne S., Leteurtre E., El Amrani M., Dharancy S., Dubuquoy L. (2021). The Impact of Modern Chemotherapy and Chemotherapy-Associated Liver Injuries (CALI) on Liver Function: Value of 99mTc-Labelled-Mebrofenin SPECT-Hepatobiliary Scintigraphy. Ann. Surg. Oncol..

[68] Zhang C., Wang H., Yang Y.H. (2026). Lipid metabolism in pancreatic cancer treatment. World J. Clin. Oncol..

[69] Lovasik B.P., Kron P., Clavien P.A., Petrowsky H., Kooby D.A. (2019). Pancreatectomy and body mass index: An international evaluation of cumulative postoperative complications using the comprehensive complications index. HPB.

[70] Zhou L., Xiao W.M., Li C.P., Gao Y.W., Gong W.J., Lu G.T. (2021). Impact of Fatty Pancreas on Postoperative Pancreatic Fistulae: A Meta-Analysis. Front. Oncol..

[71] Takemura N., Saiura A., Koga R., Yamamoto J., Yamaguchi T. (2017). Risk Factors for and Management of Postpancreatectomy Hepatic Steatosis. Scand. J. Surg..

[72] Pecorelli N., Carrara G., De Cobelli F., Cristel G., Damascelli A., Balzano G., Beretta L., Braga M. (2016). Effect of sarcopenia and visceral obesity on mortality and pancreatic fistula following pancreatic cancer surgery. Br. J. Surg..

[73] Kato H., Kamei K., Suto H., Misawa T., Unno M., Nitta H., Satoi S., Kawabata Y., Ohtsuka M., Rikiyama T. (2022). Incidence and risk factors of nonalcoholic fatty liver disease after total pancreatectomy: A first multicenter prospective study in Japan. J. Hepatobiliary Pancreat. Sci..

[74] Sugumar K., Naik L., Hue J.J., Ammori J.B., Hardacre J.M., Ocuin L.M., Winter J.M. (2024). Risk factors of developing nonalcoholic fatty liver disease after pancreatic resection: A systematic review and meta-analysis. J. Gastrointest. Surg..

[75] Huang K., Qian T., Chen W., Bai X., Gao S., Shen Y., Zhang M., Ma T., Liang T. (2024). Clinical Significance and Risk Factors of Nonalcoholic Fatty Liver Diseases After Whipple Procedure. J. Surg. Res..

[76] Okamura Y., Sugimoto H., Yamada S., Fujii T., Nomoto S., Takeda S., Kodera Y., Nakao A. (2012). Risk factors for hepatic steatosis after pancreatectomy: A retrospective observational cohort study of the importance of nutritional management. Pancreas.

[77] Fujii T., Iizawa Y., Kobayashi T., Hayasaki A., Ito T., Murata Y., Tanemura A., Ichikawa Y., Kuriyama N., Kishiwada M. (2024). Radiomics-based prediction of nonalcoholic fatty liver disease following pancreatoduodenectomy. Surg. Today.

[78] Kato H., Isaji S., Azumi Y., Kishiwada M., Hamada T., Mizuno S., Usui M., Sakurai H., Tabata M. (2010). Development of nonalcoholic fatty liver disease (NAFLD) and nonalcoholic steatohepatitis (NASH) after pancreaticoduodenectomy: Proposal of a postoperative NAFLD scoring system. J. Hepatobiliary Pancreat. Sci..

[79] Shah P., Patel V., Ashkar M. (2022). *De novo* non-alcoholic fatty liver disease after pancreatectomy: A systematic review. World J. Clin. Cases.

[80] Vujasinovic M., Demir I.E., Marchegiani G., Hegyi P., Archibugi L., Valente R., Capurso G., Witt H., Bonovas S., Piovani D. (2026). International Multidisciplinary Consensus Report on Definitions, Diagnostic Criteria, and Management of Fatty Pancreas: A Joint Statement Endorsed by EPC, APA, EASD, EASL, ESGAR, ESGE, ESP, ESPCG, ESPEN, ESPGHAN, IAP, JPS, KPBA, LAPSG, and UEG. United Eur. Gastroenterol. J..

[81] Wagner R., Eckstein S.S., Yamazaki H., Gerst F., Machann J., Jaghutriz B.A., Schürmann A., Solimena M., Singer S., Königsrainer A. (2022). Metabolic implications of pancreatic fat accumulation. Nat. Rev. Endocrinol..

[82] Mahyoub M.A., Elhoumed M., Maqul A.H., Almezgagi M., Abbas M., Jiao Y., Wang J., Alnaggar M., Zhao P., He S. (2023). Fatty infiltration of the pancreas: A systematic concept analysis. Front. Med..

[83] Lee J.S., Kim S.H., Jun D.W., Han J.H., Jang E.C., Park J.Y., Son B.K., Kim S.H., Jo Y.J., Park Y.S. (2009). Clinical implications of fatty pancreas: Correlations between fatty pancreas and metabolic syndrome. World J. Gastroenterol..

[84] van Geenen E.J., Smits M.M., Schreuder T.C., van der Peet D.L., Bloemena E., Mulder C.J. (2010). Nonalcoholic fatty liver disease is related to nonalcoholic fatty pancreas disease. Pancreas.

[85] Wongtrakul W., Untaaveesup S., Pausawadi N., Charatcharoenwitthaya P. (2023). Bidirectional association between non-alcoholic fatty liver disease and fatty pancreas: A systematic review and meta-analysis. Eur. J. Gastroenterol. Hepatol..

[86] Rosenblatt R., Mehta A., Snell D., Hissong E., Kierans A.S., Kumar S. (2019). Ultrasonographic Nonalcoholic Fatty Pancreas Is Associated with Advanced Fibrosis in NAFLD: A Retrospective Analysis. Dig. Dis. Sci..

[87] Sun Y., Zhang L., Huang J.Q., Su J., Cui L.G. (2024). Non-invasive diagnosis of pancreatic steatosis with ultrasound images using deep learning network. Heliyon.

[88] Zhou Y., He C., Zhang H. (2025). Correlation study of fatty pancreas and fatty liver CT manifestations with biochemical parameters in middle-aged and young adult cholecystectomy patients. BMC Gastroenterol..

[89] Sepe P.S., Ohri A., Sanaka S., Berzin T.M., Sekhon S., Bennett G., Mehta G., Chuttani R., Kane R., Pleskow D. (2011). A prospective evaluation of fatty pancreas by using EUS. Gastrointest. Endosc..

[90] Idilman I.S., Tuzun A., Savas B., Elhan A.H., Celik A., Idilman R., Karcaaltincaba M. (2015). Quantification of liver, pancreas, kidney, and vertebral body MRI-PDFF in non-alcoholic fatty liver disease. Abdom. Imaging.

[91] Kiemen A.L., Dbouk M., Diwan E.A., Forjaz A., Dequiedt L., Baghdadi A., Madani S.P., Grahn M.P., Jones C., Vedula S. (2024). Magnetic Resonance Imaging-Based Assessment of Pancreatic Fat Strongly Correlates with Histology-Based Assessment of Pancreas Composition. Pancreas.

[92] Arab J.P., Díaz L.A., Rehm J., Im G., Arrese M., Kamath P.S., Lucey M.R., Mellinger J., Thiele M., Thursz M. (2025). Metabolic dysfunction and alcohol-related liver disease (MetALD): Position statement by an expert panel on alcohol-related liver disease. J. Hepatol..

[93] Kalligeros M., Vassilopoulos A., Vassilopoulos S., Victor D.W., Mylonakis E., Noureddin M. (2024). Prevalence of Steatotic Liver Disease (MASLD, MetALD, and ALD) in the United States: NHANES 2017-2020. Clin. Gastroenterol. Hepatol..

[94] Johansen S., Thiele M., Krag A. (2025). Non-invasive tests for MetALD and alcohol-related liver disease. JHEP Rep..

[95] Staufer K., Huber-Schönauer U., Strebinger G., Pimingstorfer P., Suesse S., Scherzer T.M., Paulweber B., Ferenci P., Stimpfl T., Yegles M. (2022). Ethyl glucuronide in hair detects a high rate of harmful alcohol consumption in presumed non-alcoholic fatty liver disease. J. Hepatol..

[96] Apte M.V., Pirola R.C., Wilson J.S. (2010). Mechanisms of alcoholic pancreatitis. J. Gastroenterol. Hepatol..

[97] Wang F., Görgülü K., Algül H., Hu L.H. (2026). The Role of Alcohol in Pancreatic Diseases: A Comprehensive Perspective. Gastroenterology.

[98] Park J.Y., Bang S., Jeon T.J., Cho J.H., Lee K.J. (2025). Risk of and factors influencing the progression from acute to recurrent acute to chronic pancreatitis. Pancreatology.

[99] European Association for the Study of the Liver (2019). EASL Clinical Practice Guidelines on nutrition in chronic liver disease. J. Hepatol..

[100] Tan J.Y.T., Cheah C.C.M., Wang Y.T., Chang P.E.J., Krishnamoorthy T.L., Tan H.K., Salazar E. (2023). Outpatient screening with the Royal Free Hospital-Nutrition Prioritizing Tool for patients with cirrhosis at risk of malnutrition. Nutrition.

[101] Jensen G.L., Cederholm T., Correia M.I.T.D., Gonzalez M.C., Fukushima R., Pisprasert V., Blaauw R., Braz D.C., Carrasco F., Jentoft A.J.C. (2025). GLIM consensus approach to diagnosis of malnutrition: A 5-year update. JPEN J. Parenter. Enteral Nutr..

[102] Tuo S., Yeo Y.H., Chang R., Wen Z., Ran Q., Yang L., Fan Q., Kang J., Si J., Liu Y. (2024). Prevalence of and associated factors for sarcopenia in patients with liver cirrhosis: A systematic review and meta-analysis. Clin. Nutr..

[103] Montano-Loza A.J., Angulo P., Meza-Junco J., Prado C.M., Sawyer M.B., Beaumont C., Esfandiari N., Ma M., Baracos V.E. (2016). Sarcopenic obesity and myosteatosis are associated with higher mortality in patients with cirrhosis. J. Cachexia Sarcopenia Muscle.

[104] Romero-Gómez M., Zelber-Sagi S., Trenell M. (2017). Treatment of NAFLD with diet, physical activity and exercise. J. Hepatol..

[105] Rivera-Esteban J., Valverde-Salazar V., Calleja J.L. (2025). Semaglutide in Metabolic Dysfunction-Associated Steatohepatitis. N. Engl. J. Med..

[106] Sualeheen A., Tan S.Y., Georgousopoulou E., Daly R.M., Tierney A.C., Roberts S.K., George E.S. (2024). Mediterranean diet for the management of metabolic dysfunction-associated steatotic liver disease in non-Mediterranean, Western countries: What’s known and what’s needed?. Nutr. Bull..

[107] Zhang S., Yan Y., Meng G., Zhang Q., Liu L., Wu H., Gu Y., Wang X., Zhang J., Sun S. (2023). Protein foods from animal sources and risk of nonalcoholic fatty liver disease in representative cohorts from North and South China. J. Intern. Med..

[108] Fu H., Li P., Sun S., Li L. (2024). Validation of the Global Leadership Initiative on Malnutrition Criteria for Predicting Adverse Outcomes in Acute Pancreatitis. Ther. Clin. Risk Manag..

[109] Arvanitakis M., Ockenga J., Bezmarevic M., Gianotti L., Krznarić Ž., Lobo D.N., Löser C., Madl C., Meier R., Phillips M. (2024). ESPEN practical guideline on clinical nutrition in acute and chronic pancreatitis. Clin. Nutr..

[110] Khurmatullina A.R., Andreev D.N., Maev I.V., Kucheryavyy Y.A., Beliy P.A., Dzhafarova A.R., Cherenkova V.V., Sokolov F.S. (2025). Prevalence and Risk of Sarcopenia in Patients with Chronic Pancreatitis: Systematic Review and Meta-Analysis. Nutrients.

[111] Bruni A., Colecchia L., Dell’Anna G., Scalvini D., Mandarino F.V., Lisotti A., Fuccio L., Cecinato P., Marasco G., Donatelli G. (2025). Nutritional Management in Chronic Pancreatitis: From Exocrine Pancreatic Insufficiency to Precision Therapy. Nutrients.

[112] Poulia K.A., Sarantis P., Antoniadou D., Koustas E., Papadimitropoulou A., Papavassiliou A.G., Karamouzis M.V. (2020). Pancreatic Cancer and Cachexia-Metabolic Mechanisms and Novel Insights. Nutrients.

[113] Láinez Ramos-Bossini A.J., Gámez Martínez A., Luengo Gómez D., Valverde-López F., Melguizo C., Prados J. (2024). Prevalence of Sarcopenia Determined by Computed Tomography in Pancreatic Cancer: A Systematic Review and Meta-Analysis of Observational Studies. Cancers.

[114] Heymsfield S.B., Aronne L.J., Montgomery P., Klickstein L.B., Coleman L.A., Dole K., Mindeholm L., Spruill S., Li X., Attie K.M. (2026). Bimagrumab plus semaglutide alone or in combination for the treatment of obesity: A randomized phase 2 trial. Nat. Med..

[115] Shimosegawa T., Chari S.T., Frulloni L., Kamisawa T., Kawa S., Mino-Kenudson M., Kim M.H., Klöppel G., Lerch M.M., Löhr M. (2011). International consensus diagnostic criteria for autoimmune pancreatitis: Guidelines of the International Association of Pancreatology. Pancreas.

[116] Lanzillotta M., Vujasinovic M., Löhr J.M., Della Torre E. (2025). Update on Autoimmune Pancreatitis and IgG4-Related Disease. United Eur. Gastroenterol. J..

[117] Rodriguez-Duque J.C., Calleja J.L., Iruzubieta P., Hernández-Conde M., Rivas-Rivas C., Vera M.I., Garcia M.J., Pascual M., Castro B., García-Blanco A. (2023). Increased risk of MAFLD and Liver Fibrosis in Inflammatory Bowel Disease Independent of Classic Metabolic Risk Factors. Clin. Gastroenterol. Hepatol..

[118] Untaaveesup S., Kantagowit P., Ungprasert P., Kitlertbanchong N., Vajiraviroj T., Sutithavinkul T., Techataweewan G., Eiumtrakul W., Threethrong R., Chaemsupaphan T. (2025). The Risk of Metabolic Dysfunction-Associated Steatotic Liver Disease in Moderate-to-Severe Psoriasis: A Systematic Review and Meta-Analysis. J. Clin. Med..

[119] Vassilopoulos A., Kalligeros M., Vassilopoulos S., Shehadeh F., Benitez G., Kaczynski M., Lazaridou I., Promrat K., Wands J.R., Mylonakis E. (2024). Prevalence of Steatotic Liver Disease Among US Adults with Rheumatoid Arthritis. Dig. Dis. Sci..

[120] Matsumoto K., Kikuchi K., Kuniyoshi N., Tsunashima H., Sekine K., Mabuchi M., Doi S., Zen Y., Miyakawa H. (2019). Immunoglobulin G4-related Liver Disease Overlapping with Non-alcoholic Steatohepatitis That Was Diagnosed Simultaneously with Autoimmune Pancreatitis: A Case Report and Review of the Literature. Intern. Med..

[121] Adams L.A., Lindor K.D., Angulo P. (2004). The prevalence of autoantibodies and autoimmune hepatitis in patients with nonalcoholic Fatty liver disease. Am. J. Gastroenterol..

[122] Stephenson A.L., Sykes J., Stanojevic S., Quon B.S., Marshall B.C., Petren K., Ostrenga J., Fink A.K., Elbert A., Goss C.H. (2017). Survival Comparison of Patients With Cystic Fibrosis in Canada and the United States: A Population-Based Cohort Study. Ann. Intern. Med..

[123] Sellers Z.M., Assis D.N., Paranjape S.M., Sathe M., Bodewes F., Bowen M., Cipolli M., Debray D., Green N., Hughan K.S. (2024). Cystic fibrosis screening, evaluation, and management of hepatobiliary disease consensus recommendations. Hepatology.

[124] Lamireau T., Monnereau S., Martin S., Marcotte J.E., Winnock M., Alvarez F. (2004). Epidemiology of liver disease in cystic fibrosis: A longitudinal study. J. Hepatol..

[125] Dana J., Debray D., Beaufrère A., Hillaire S., Fabre M., Reinhold C., Baumert T.F., Berteloot L., Vilgrain V. (2022). Cystic fibrosis-related liver disease: Clinical presentations, diagnostic and monitoring approaches in the era of CFTR modulator therapies. J. Hepatol..

[126] Kutney K., Donnola S.B., Flask C.A., Gubitosi-Klug R., O’Riordan M., McBennett K., Sferra T.J., Kaminski B. (2019). Lumacaftor/ivacaftor therapy is associated with reduced hepatic steatosis in cystic fibrosis patients. World J. Hepatol..

[127] Gardner T.B., Park W.G., Allen P.J. (2024). Diagnosis and Management of Pancreatic Cysts. Gastroenterology.

[128] Sbeit W., Greener T., Kadah A., Mari A., Goldin E., Khoury T., Mahamid M. (2021). Pancreatobiliary manifestations of nonalcoholic fatty liver disease: A retrospective case-control multicenter study. Eur. J. Gastroenterol. Hepatol..

[129] Tanaka S., Tsujimae M., Masuda A., Inoue J., Inomata N., Uemura H., Kohashi S., Nagao K., Masuda S., Abe S. (2024). Metabolic Syndrome Accelerates the Age-Related Increase of Intraductal Papillary Mucinous Neoplasm of the Pancreas. Pancreas.

[130] Davies N.M., Holmes M.V., Davey Smith G. (2018). Reading Mendelian randomisation studies: A guide, glossary, and checklist for clinicians. BMJ.

[131] Hernán M.A., Robins J.M. (2016). Using Big Data to Emulate a Target Trial When a Randomized Trial Is Not Available. Am. J. Epidemiol..

[132] Lipsitch M., Tchetgen Tchetgen E., Cohen T. (2010). Negative controls: A tool for detecting confounding and bias in observational studies. Epidemiology.

[133] Elshaer A., Chascsa D.M.H., Lizaola-Mayo B.C. (2024). Exploring Varied Treatment Strategies for Metabolic Dysfunction-Associated Steatotic Liver Disease (MASLD). Life.

[134] Liao C., Liang X., Zhang X., Li Y. (2023). The effects of GLP-1 receptor agonists on visceral fat and liver ectopic fat in an adult population with or without diabetes and nonalcoholic fatty liver disease: A systematic review and meta-analysis. PLoS ONE.

